# MicroRNA and MicroRNA-Target Variants Associated with Autism Spectrum Disorder and Related Disorders

**DOI:** 10.3390/genes13081329

**Published:** 2022-07-26

**Authors:** Anthony Wong, Anbo Zhou, Xiaolong Cao, Vaidhyanathan Mahaganapathy, Marco Azaro, Christine Gwin, Sherri Wilson, Steven Buyske, Christopher W. Bartlett, Judy F. Flax, Linda M. Brzustowicz, Jinchuan Xing

**Affiliations:** 1 Department of Genetics, Rutgers, The State University of New Jersey, Piscataway, NJ 08854, USA; aw449@scarletmail.rutgers.edu (A.W.); zhouanbo@gmail.com (A.Z.); atps@outlook.com (X.C.); vaidhyanathan.m@rutgers.edu (V.M.); azaro@hginj.rutgers.edu (M.A.); gwin@dls.rutgers.edu (C.G.); sherriw@hginj.rutgers.edu (S.W.); judyflax17@gmail.com (J.F.F.); lbrz@hginj.rutgers.edu (L.M.B.); 2 Department of Statistics, Rutgers, The State University of New Jersey, Piscataway, NJ 08854, USA; buyske@stat.rutgers.edu; 3 The Steve & Cindy Rasmussen Institute for Genomic Medicine, Battelle Center for Computational Biology, Abigail Wexner Research Institute at Nationwide Children’s Hospital, Columbus, OH 43215, USA; christopher.bartlett@nationwidechildrens.org; 4 Department of Pediatrics, College of Medicine, The Ohio State University, Columbus, OH 43210, USA; 5 Human Genetics Institute of New Jersey, Rutgers, The State University of New Jersey, Piscataway, NJ 08854, USA

**Keywords:** whole-genome sequencing, miRNA, autism spectrum disorder, family cohort, 3′ UTR, neurodevelopmental disorder

## Abstract

Autism spectrum disorder (ASD) is a childhood neurodevelopmental disorder with a complex and heterogeneous genetic etiology. MicroRNA (miRNA), a class of small non-coding RNAs, could regulate ASD risk genes post-transcriptionally and affect broad molecular pathways related to ASD and associated disorders. Using whole-genome sequencing, we analyzed 272 samples in 73 families in the New Jersey Language and Autism Genetics Study (NJLAGS) cohort. Families with at least one ASD patient were recruited and were further assessed for language impairment, reading impairment, and other associated phenotypes. A total of 5104 miRNA variants and 1,181,148 3′ untranslated region (3′ UTR) variants were identified in the dataset. After applying several filtering criteria, including population allele frequency, brain expression, miRNA functional regions, and inheritance patterns, we identified high-confidence variants in five brain-expressed miRNAs (targeting 326 genes) and 3′ UTR miRNA target regions of 152 genes. Some genes, such as *SCP2* and *UCGC*, were identified in multiple families. Using Gene Ontology overrepresentation analysis and protein–protein interaction network analysis, we identified clusters of genes and pathways that are important for neurodevelopment. The miRNAs and miRNA target genes identified in this study are potentially involved in neurodevelopmental disorders and should be considered for further functional studies.

## 1. Introduction

Autism spectrum disorder (ASD) is a multifaceted neurodevelopmental disorder (NDD) identified by neurodevelopmental impairment, deficits in social communication, and repetitive patterns of behavior [1]. Published studies by the Center for Disease Control and Prevention (CDC) and the New Jersey Autism Study showed alarming rates of ASD prevalence in children, i.e., 1 in 59 nationally [2]. The high prevalence of ASD has made it essential to study the biological mechanism of, and identify predictive biomarkers for, the disorder.

ASD is highly heritable, with a recurrence rate around 20% among family members [3]. Previous studies of the genetic etiology of ASD have identified a large number of genetic risk factors that contribute to the disorder. These risk factors can be broadly divided into three classes: (1) small genetic variants (i.e., single nucleotide variants (SNVs) and insertion/deletion variants (indels)) in the protein coding regions and non-coding regions (~50% of ASD cases) [4,5]; (2) rare copy-number variants (5–10% of ASD cases) and chromosomal abnormalities (~5% of ASD cases); and (3) ASD-related genetic syndromes (~10% of cases) [4]. At the gene level, nearly 1000 genes are implicated in ASD. Although the genome-wide genetic risk based on common variants can be estimated for a patient, due to the heterogeneity of ASD, each identified mutation contributes only a small percentage of known cases, and in each patient, the combination of variants that resulted in ASD is mostly undetermined.

MicroRNAs (miRNAs) are a type of short, non-coding genes that are involved in post-transcriptional gene regulation. A single miRNA can bind to several hundred target sites in the 3′ untranslated regions (3′ UTR) of mRNAs and thus control a large variety of molecular pathways that could be involved in ASD pathophysiology. Indeed, miRNAs are expressed in different brain regions and have crucial functions during brain development [6]. Previous studies of dysregulated miRNAs identified several candidates [7,8]. For example, a study using mouse neurons discovered that miR-137 and miR-132 regulate two autism-related genes: *PTEN* and *MeCP2* [7]. A study of model mice with Angelman Syndrome discovered down-regulation of miR-708 in the brain, which led to aberrant Ca2+ signaling [8]. Thus, miRNA dysfunction could play a role in explaining the complex and heterogeneous nature of ASD and related NDDs.

While there are previous whole-genome sequencing (WGS) studies of miRNA mutations and their target regions in ASD patients [9,10], few if any studies have quantified the relationship between putative genes in ASD and other associated NDDs in a familial setting. In this study, we utilized WGS data from the New Jersey Language and Autism Genetics Study (NJLAGS) to examine the contribution of miRNAs to ASD and related disorders. NJLAGS is designed to detect genetic variation related to both ASD and language impairment [11]. More than 150 families were recruited in NJLAGS and this cohort provides the architecture to assess the contribution of both segregating and *de novo* variants to ASD genetic etiology. Here, we identified high-confidence SNVs and indels in miRNA and miRNA target regions from the NJLAGs cohort. We propose that mutations that affect miRNAs and their mRNA transcript binding sites could cause abnormal gene expression for ASD implicated genes and contribute to ASD and related NDDs in NJLAGS families.

## 2. Materials and Methods

### 2.1. Family Selection and Phenotyping

NJLAGS collected data from ASD probands and their family members for genetic analysis. Each individual was subjected to measures of ASD, oral language impairment (LI), written language impairment (RI), and social responsiveness (SRS). The assessment and diagnosis details were described previously [11]. Briefly, ASD diagnoses were based on the Autism Diagnostic Interview (ADI-R), Autism Diagnostic Observation Schedule (ADOS), and the Diagnostics and Statistical Manual-IV (DSM-IV), or DSM-5, depending on the recruitment date (see [11] for details). LI is defined as a score of less than 85 on the CELF-4 or a history of language/reading difficulties with at least a score of one standard deviation below their peers on at least 60% of oral language subtests. RI is defined as a score of one standard deviation below the mean of 60% on all reading tests. SRS is measured with the Social Responsiveness Scale. The cut-off for a dichotomous trait social deficit (SRS-DT) was a score of 54 for males and 45 for females which was equivalent to the T-score > 60 criteria used to identify mild impairment in children [11]. ADHD affection status for ASD probands and their family members was determined based on the relevant questionnaires, as described previously [12]. This study was approved by the Institutional Review Board at Rutgers, State University of New Jersey (IRB number: 13-112Mc).

### 2.2. miRNA Variant Identification

A total of 272 individuals were subject to WGS (Table S1). The details of sequencing and variant identification are described previously [12]. The SNVs and indels were obtained from a previous study [12] and are available in the National Institute of Mental Health Data Archive (NDA) under experiments C1932 and C2933.

The genomic positions of human miRNA genes (genome assembly GRCh37) were obtained from miRBase v22 [13]. The database includes 1917 miRNA genes in the human genome [14]. For variants within a known miRNA, ADmiRE [15,16] was used to classify the variants by their locations in miRNA functional regions (i.e., seed, mature, etc.). The seed region annotation indicates a variant lies within the core miRNA/mRNA binding region, while the mature region indicates a variant lies outside of the seed region but still within a functionally mature miRNA that is incorporated into the RNA-induced silencing complex. The arm and loop regions form the remaining hairpin of the precursor miRNA structure.

### 2.3. MiRNA Binding Site Identification

TargetScanHuman [17] was used to predict potential miRNA target genes [18]. Binding sites are categorized as broadly conserved across vertebrate species, conserved across mammals, or poorly conserved by aligning sequences across 28 different species [19]. Broadly conserved and conserved binding sites were considered high-confidence candidates and selected for downstream analysis. Using this data, 3′ UTR variants were overlapped with TargetScanHuman conserved binding sites to identify 3′ UTR variants that could disrupt miRNA-mRNA binding. To characterize the gene networks regulated by candidate miRNAs, miRNA predicted targets with a context++ score (CS) of < −0.4 was used to select sites with high targeting efficacy [18].

### 2.4. Characterizing Variants by Inheritance Modes

GEMINI v0.20.1 [20] is a computational framework that identifies variants following autosomal dominant, autosomal recessive, or *de novo* mode of inheritance [21]. Both pedigree and variant call information was loaded into GEMINI using the following command:

*gemini load -v input.vcf -p input.ped*

Variants were then identified as being autosomal dominant, autosomal recessive, or *de novo* for each phenotype by executing:

*gemini autosomal_dominant/autosomal_recessive/de_novo input.db*

GEMINI strict inheritance rules were applied, where at least a full trio (i.e., both parents are present for a proband) is required to infer the inheritance pattern.

### 2.5. Reference Population Dataset

WGS data from 15,708 (gnomAD v.2.1.1 non-neuro genome, Broad Institute, Cambridge, MA, USA) and 76,156 (gnomAD v.3 non-neuro, Broad Institute, Cambridge, MA, USA) unrelated individuals without a neurological condition, and whole-exome sequencing data from 125,748 unrelated individuals without a neurological condition (gnomAD v.2.1.1 non-neuro exome, Broad Institute, Cambridge, MA, USA) were obtained from the Genome Aggregation Database (gnomAD) [22] and used as reference populations. Variants that failed gnomAD quality control metrics (i.e., with RF, AC0, InbreedingCoeff, etc. in the “Filters” field) were excluded. Variant alternative allele frequencies (AF) in gnomAD populations were used to identify rare alleles in the general population. An AF cutoff of 1% for dominant and recessive and 0.1% for *de novo* variants in all datasets was applied for miRNA and 3′ UTR variants. For variants which do not have AF values in all gnomAD datasets, the AF cutoffs were applied based on available databases. Variants that are not identified in gnomAD databases were instead filtered by cohort AF among the NJLAGS samples (1% for dominant and recessive and absent in all other samples for *de novo* variants).

### 2.6. Brain Expression Data

Expressions of miRNA and protein-coding genes in the brain were extracted from the Gene Tissue Expression project (GTEx) [23], the BrainSpan Atlas of the Developing Human Brain project [24], and the Human Developmental Biology Resource (HDBR) [25]. Detailed data processing procedures were described previously [26]. We selected genes and miRNAs that have a transcript per million (TPM) value greater than 5 in any of the datasets.

### 2.7. Gene Ontology Enrichment Analysis and Protein–Protein Interaction Network Analysis

Gene Ontology (GO) enrichment analysis was performed using ConsensusPathDB [27]. GO terms with a false-discovery rate (FDR) < 0.05 were considered enriched and terms with less than 600 total genes within the group were selected to increase the specificity of the enrichment results.

A protein–protein interaction network was built to investigate the potential interactions between candidate miRNA target genes and 3′ UTR genes. ConsensusPathDB [27], STRING [28], and GIANT_v2 [29] were used to generate a list of known gene interactions. Detailed data processing procedures were described previously [26,30]. Edges with at least two database interactions were considered high-confidence interactions for the network. Additional known NDD genes collected from previous studies (Table S2, [26]) were included in the network analysis. Isolated and self-directed interactions were removed from the network.

## 3. Results

### 3.1. Cohort Description

We obtained WGS variants of 272 individuals from 73 families (Table S1). 65 males and 18 females were diagnosed with ASD (Table 1). All 73 families contained at least one individual diagnosed with ASD, LI, or RI, and 59 and 47 families contained at least one individual diagnosed with SRS and ADHD. Across all disorders, 35 and 67 families fit a dominant or a recessive/*de novo* mode of inheritance, respectively (Table 1). Due to a lack of ASD affected parents in the cohort of sequenced individuals, no families met the criteria for dominant inheritance pattern for ASD.

### 3.2. miRNA Variant Identification

**Figure 1:**
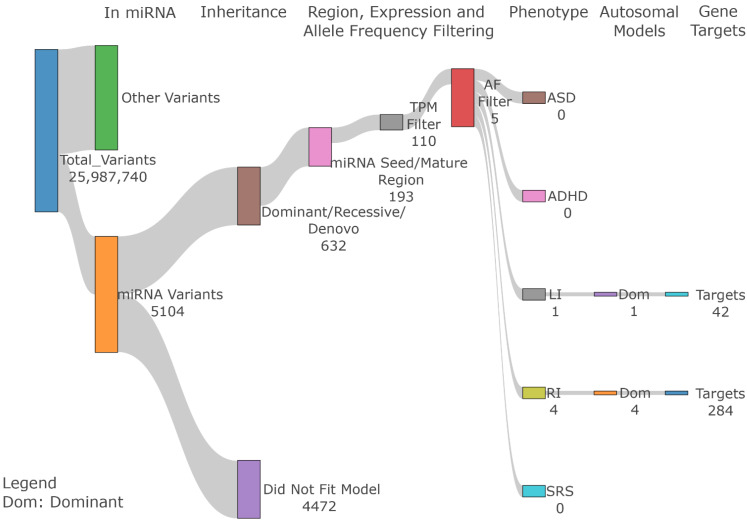
**miRNA variant filtering steps.** After selecting all variants within miRNAs, we filtered variants based on their inheritance pattern, miRNA region annotation, brain expression pattern, and population allele frequency to select final candidate variants.

Out of 25,987,740 variants, 5104 variants overlapped miRNA genes and were annotated for the miRNA regions (e.g., seed, mature, etc.). Using the GEMINI program, we categorized the variants by their inheritance pattern for each phenotype. A total of 632 variants matched one of the segregation patterns (dominant, recessive, or *de novo*) (Figure 1, see Table S3 for details). Among them, we selected the 193 miRNA variants in seed and mature regions for downstream analysis, because variants in mature miRNAs and seed regions are most likely to affect the miRNA function. Among the 193 variants, 110 are within miRNAs expressed in the brain (see Methods for detail). To select rare variants, we applied AF filtering of less than 1% for dominant and recessive variants and 0.1% for *de novo* variants in individuals in the gnomAD database. After filtering, five unique miRNA variants from four families meet the AF cutoff under the dominant mode of inheritance (Table 2).

Out of the five variants, two variants, rs371749301 and rs550720421, were identified in family FAM58 and segregated with the RI phenotype. Two additional variants (rs200279579, rs761222509) segregated with RI in families FAM5 and FAM66 and one segregated with LI (rs565141718) in family FAM13, respectively (Table 2).

### 3.3. 3′ UTR miRNA Binding Site Variant Identification

**Figure 2:**
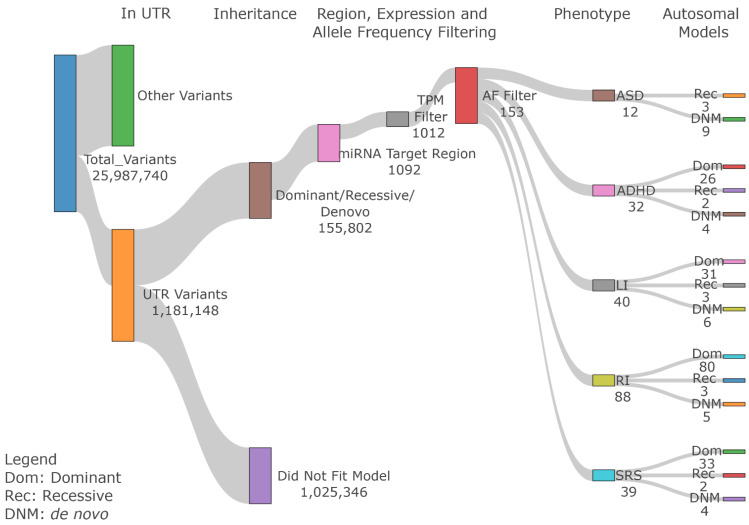
**3′ UTR variant filtering steps.** After selecting all variants within 3′ UTR regions, we filtered variants based on their inheritance pattern, miRNA target region annotation, brain expression pattern, and population allele frequency to select final candidate variants.

**Figure 3:**
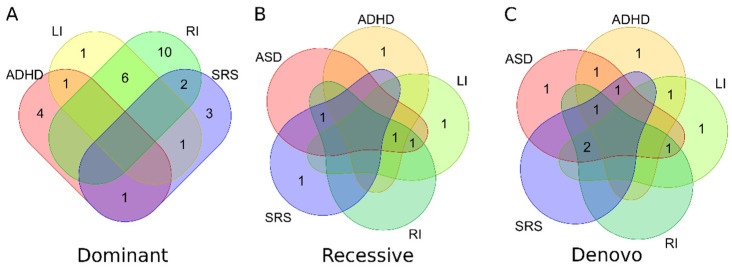
**Number of families with 3′ UTR variants.** Venn Diagrams of families with 3′ UTR variants for each inheritance pattern: (**A**) Dominant; (**B**) Recessive; (**C**) *de novo*. The value indicates the number of families with candidate variants for the phenotype(s).

In addition to miRNA variants, variants in miRNA binding sites in the 3′ UTR of miRNA target genes can also affect miRNA regulation. Next, we identified variants in the miRNA binding sites. In the dataset, 1,181,148 variants were identified within 3′ UTR regions. These variants were subject to similar variant filtering criteria as miRNA variants (Figure 2). Using GEMINI, 155,802 variants fell within one of the three inheritance pattern categories. TargetscanHuman was then used to identify 1092 variants that overlapped predicted miRNA conserved target sites (context++ score < −0.4). Applying an AF filter of 1% for dominant and recessive variants and 0.1% for *de novo* variants resulted in 153 unique variants in 152 brain-express genes (Table 3), see Supplemental Table S4 for details). A total of 6, 9, 18, and 7 families containing variants segregate in the dominant pattern for ADHD, LI, RI, and SRS, respectively (Figure 3A). Because some patients have multiple diagnoses, some variants meet certain segregation pattern in multiple phenotypes (Table S4). Therefore, 29, 6, and 10 unique families contain variants segregating in dominant, recessive, and *de novo* patterns, respectively (Figure 3).

### 3.4. Candidate Gene Analysis

To understand the functional impact of variants in miRNAs and their binding sites on genes and molecular pathways, we first identified target genes of segregating miRNA variants. We selected genes that are expressed in brain tissues for their biological relevance (see Methods for detail). RI had four miRNAs with 284 target genes, and LI had one miRNA with 42 target genes (Table S5). Next, we selected brain-expressed genes containing segregating variants in the 3′ UTR miRNA binding sites. A total of 12, 32, 39, 87, and 39 unique genes were selected for ASD, ADHD, LI, RI, and SRS, respectively (Table 3 and Table S5). 

Four genes, *ONECUT2*, *OSBP*, *SCP2*, and *TUB*, appeared as targets of candidate miRNAs and contained variants in their 3′ UTR miRNA target region (Table 4). For example, Family FAM5 carried a mutation within the seed region of hsa-miR-6780a-3p (rs200279579; chr17:40860121-A-G) which targets *SCP2* (Sterol Carrier Protein 2). Family FAM2 contained a mutation within the *SCP2* 3′ UTR region (rs182947399; chr1:53516762-A-T) which affects the *SCP2*/miR-150-5p binding axis. Both mutations segregate in a dominant pattern with the RI phenotype. 

Two candidate genes, *RBM24* and *UGCG*, overlapped multiple phenotypes in more than one family (Table 4). *UGCG* (UDP-Glucose Ceramide Glucosyltransferase) is a key gene for the biosynthesis of glycosphingolipids. The dominant variant (rs201977317; chr9:114695431-GA-G) appeared in family FAM5 for RI and families FAM5, FAM36, and FAM37 for SRS. The importance of *UGCG* and the role of glycosphingolipids in brain development has been reported previously [31,32].

**Table 4:** Top Candidate Genes.

Gene					miRNA Variant				3′ UTR Variant						
Gene	Max Brain Expression	Other NDD	pLI	Previous Studies	miRNA	Inheritance	Family	Phenotype	chr	pos	rsID	AF_gnomAD	Inheritance	Family	Phenotype
*ONECUT2*	66	NA	0.91	[33]	hsa-miR-6780a-3p	dominant	FAM5	RI	chr18	55155836	rs147208471	7.27 × 10^−4^	dominant	FAM56	SRS
*OSBP*	61.03	NA	1.00	[34]	hsa-miR-6780a-3p	dominant	FAM5	RI	chr11	59343007	rs149325846	3.61 × 10^−3^	dominant	FAM14; FAM66	RI
*SCP2*	65.43	ASD_Low	0.00	[35]	hsa-miR-6780a-3p	dominant	FAM5	RI	chr1	53516762	rs182947399	1.33 × 10^−3^	dominant	FAM2	ADHD
*TUB*	187	NA	0.00	[36,37]	hsa-miR-6780a-3p	dominant	FAM5	RI	chr11	8123523	rs1379616749	3.12 × 10^−4^	dominant	FAM70	RI
*RBM24*	49.40	NA	0.09	[38]					chr6	17292448	rs914886490	2.60 × 10^−4^	*de novo*	FAM5; FAM59	ADHD,LI
*UGCG*	86	NA	0.94	[31,32]					chr9	114695431	rs201977317	4.54 × 10^−3^	dominant	FAM5; FAM36; FAM37	RI,SRS

Other NDD: genes implicated in NDDs from previous studies (Table S2). pLI: the probability the gene is loss-of-function intolerant. AF_gnomAD: maximum variant AF in the three gnomAD databases. Other headers are the same as in Table 2.

### 3.5. Gene Ontology Terms Early Forebrain Patterning and Apoptotic Process Involved in Development Are Enriched

To explore the possible shared relationship and affected pathways between our two sets of genes, we performed a GO over-representation analysis to identify neurodevelopmental terms in each phenotype (Table S6). Each gene list showed enrichment in pathways and terms related to brain structure and development. For example, “Forebrain dorsal/ventral pattern formation” (GO:0021798, q = 0.0038) is a small category consisting of five genes, three of which (*GSX2, SIX3, NKX2-1*) appear in our RI candidate genes. Other top terms include “apoptotic process involved in development” (GO:1902742, q = 0.003) and “actin cytoskeleton” (GO:0015629, q = 0.038) in LI and ASD candidate genes, respectively, and may suggest the importance of the regulation of apoptosis and actin in the context of neurodevelopment.

### 3.6. Protein–Protein Interactions

**Figure 4:**
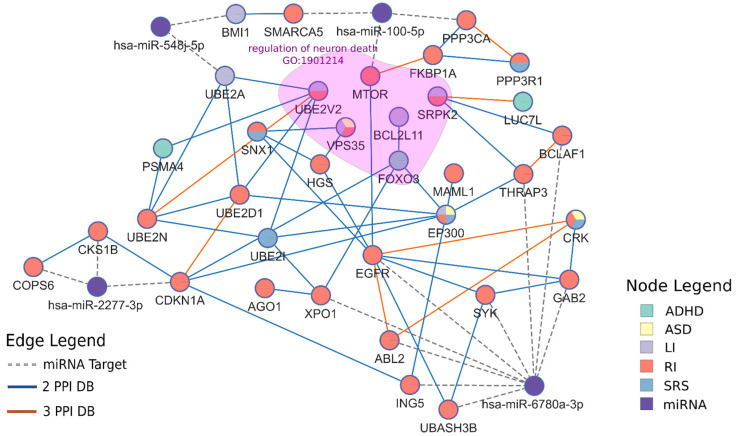
**Candidate gene PPI network.** The network includes both miRNA target genes and 3′ UTR genes (Table S9). PPI edges are included if at least two databases show evidence of interaction. The highlighted region included genes enriched in regulation of neuron death (GO:1901214).

A protein–protein interaction (PPI) network can help identify shared etiology among families despite the heterogeneity of single gene variants among individuals. Therefore, we constructed a PPI network for both miRNA target genes and 3′ UTR genes. Seventeen out of 327 miRNA target genes and 18 out of 152 3′ UTR genes have high-confidence interactions within a single network (Figure 4, Table S7). An enrichment analysis on the 35 connected genes showed enrichment in terms such as “regulation of neuron death” (GO:1901214, q = 1.21 × 10^−4^). While these genes are not directly connected, it demonstrates a control of neuronal development from many different pathways that our candidate genes may be involved in. One of our top terms was “calcium-mediated signaling” (GO:0019722, q = 2.23 × 10^−5^) and overlapped 6 genes. Other terms are listed in Table S8. In addition, five genes, *MTOR, AGO1, EP300, XPO1, and PPP3CA*, have been implicated in ASD by the SFARI database. *AGO1* (Argonaute RISC Component 1), is the protein directly involved in the miRNA-mRNA binding complex and lends further credence to miRNA involvement in ASD and related NDDS.

**Figure 5:**
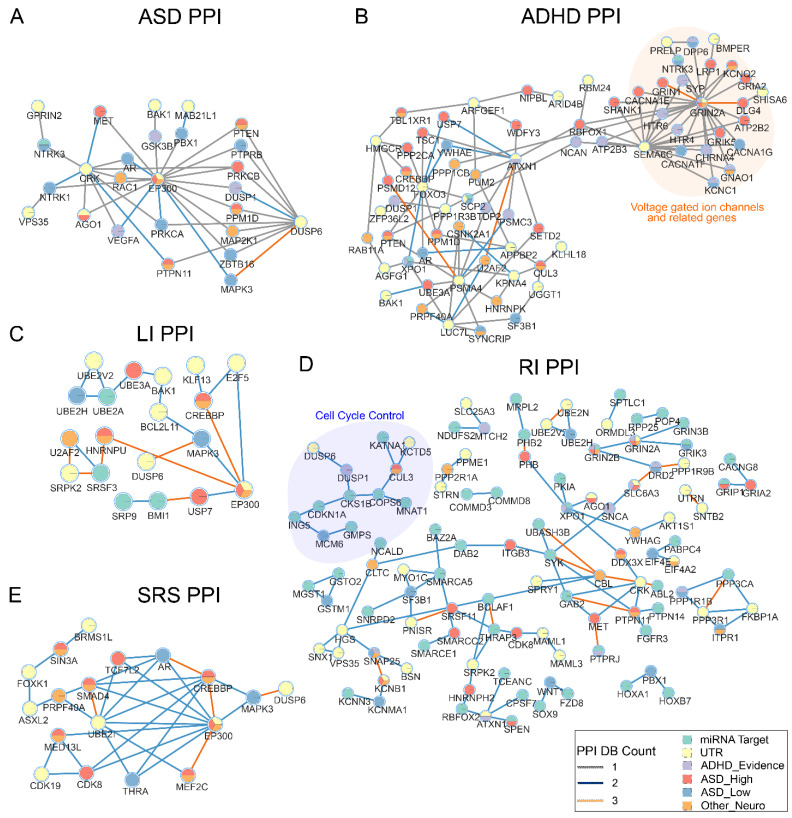
**Candidate gene interaction network with known NDD genes.** Known genes implicated in ASD, ADHD, and other NDDs (Table S2) were included in the PPI network with candidate genes in this study. (**A**) ASD candidates PPI. (**B**) ADHD candidates PPI. (**C**) LI candidates PPI. (**D**) RI candidates PPI. Nodes with degree greater than 13 were hidden from the network for clarity. (**E**) SRS candidates PPI. Isolated nodes were removed from the networks. Connections between non-candidate genes were not shown. Edges with only one protein–protein database evidence were not shown in (**C**,**E**). Highlighted regions included genes in GO terms.

We then constructed additional networks between our candidate genes and known NDD genes to further explore the effect of our genes in NDDs (Figure 5, Table S10). The ADHD network (Figure 5B) shows a tightly clustered set of calcium voltage gated and glutamate receptor genes, both of which have been shown to be possible mechanisms linked to ASD and ADHD [39]. The RI and LI PPI networks contain genes involved in chromatin and transcription factor binding and cell cycle control (Figure 5C,D). Common genes that appear in multiple networks include *MAPK3* and *PTEN*. *MAPK3* is located within the chromosome 16p11.2 band that is associated with 16p11.2 deletion syndrome, characterized by intellectual disability and developmental delay [40]. *PTEN* has been reported to be involved in a miRNA regulatory network and mutations in *PTEN* are present in 20% of children with both ASD and macrocephaly [7,41]. Candidate genes also interact with *NTRK1* and *NTRK3*, Neurotrophic Receptor Tyrosine Kinases, which interact with nerve growth factors.

## 4. Discussion

The genetic etiology of ASD has been studied extensively; however, many ASD patients have no known genetic cause [4]. Due to the role of miRNA in brain development, variants in miRNAs and their binding targets in 3′ UTRs could contribute genetically to the emergence of ASD. In this study, we sought to identify candidate genes affected by miRNA/mRNA interaction variants by leveraging the WGS data generated from families collected by NJLAGS.

By studying segregating variants that were also rare in the general population, we identified five miRNA and 153 3′ UTR high-confidence variants in 39 families across the five phenotypes. Although the large number of variants prevented us from performing experimental validation, all five miRNA candidate variants and the vast majority of the 3′ UTR variants (146 out of 153) were either reported as high-quality variants in the gnomAD project or in multiple samples in our dataset. Combining with our stringent genotype segregation screening, we expect that most of the candidate variants are authentic. These variants are located in the seed/mature region of miRNAs or in conserved 3′ UTR miRNA target sites and have a high probability of disrupting the complementary base pairing in the miRNA-mRNA complex. Six genes (Table 4) have high-confidence variants in multiple families, making them strong candidates as risk genes. *TUB* (TUB Bipartite Transcription Factor) and *SCP2* have been studied in individuals with neuronal disorders [35,36]. *TUB* is part of the Tubby signaling pathways that are important during neurodevelopment [37]. A *TUB* frameshift mutation in three siblings was associated with retinal dystrophy and obesity [36]. *SCP2* is responsible for mediating the transfer of common phospholipids, cholesterol, and gangliosides, and is implicated in Leukoencephalopathy with Dystonia and Motor Neuropathy [35]. For the remaining genes, in vivo studies provide evidence of their role in neuronal development. *ONECUT2* (One Cut Homeobox 2) is known to regulate early retinal progenitor cells [33]. A study of N2A mice neuroblast cell lines discovered that the overexpression of *OSBP* (Oxysterol Binding Protein) decreased miR-124 mediated neurite growth [34]. *RBM24* (RNA Binding Motif Protein 24) is an RNA-binding protein that is responsible for skeletal myogenesis, heart development, vertebrae sensory organ differentiation, and embryonic germ layer formation [38]. *UGCG* (UDP-Glucose Ceramide Glucosyltransferase) is important in the neural differentiation process [31]. *Ugcg* inhibition in embryonic mouse cell lines was found to decrease neural cell marker proteins *GFAP* and *MAP-2* [31]. While the six candidate genes appear in different processes, possibly due to the heterogeneity of the disorders, previous studies have shown that each gene plays important roles during neuronal development, and the disruption of their expression regulation could have contributed to the disorders in these families.

As expected with the heterogeneity of ASD, our study found hundreds of genes that were unique to single families. To further elucidate the contribution of these candidate genes, we conducted functional enrichment analyses and constructed PPI networks with known NDD genes. The results showed that our miRNA target genes are involved in processes which are important to neurodevelopment. For example, the ASD phenotype showed enrichment of the GO term “actin cytoskeleton”, which is a component of dendritic spine formation and plasticity [42]. A study of stem cells from human exfoliated deciduous teeth of 13 ASD patients showed impaired actin polymerization in ASD patients and demonstrated a possible mechanism of NDDs [43]. Three genes from our RI group, *GSX2*, *SIX3*, and *NKX2-1*, are known to be expressed in the early forebrain and are responsible for forebrain dorsal-ventral pattern formation during early cortical development [44,45,46], an important developmental stage for ASD [47]. Additional functional analysis of our 37 genes in a PPI network (Figure 4) resulted in an enrichment of regulation of neuron death. Neuronal apoptosis is an important mechanism during neurodevelopment and has been implicated in ASD. For example, a comparison of neuron counts of seven autistic and six control children found 67% more neurons in the prefrontal cortex in autistic children than the mean. Additionally, a failure of subplate apoptosis in the prenatal brain is proposed as one reason for increase neuron counts in ASD children [48]. A look into our phenotype subgraphs (Figure 5) reveals that our candidate genes target some key autism risk genes. For example, *PTEN* appears to be strongly connected in our network and has appeared as a high confidence risk gene for individuals with ASD and macrocephaly [49,50,51]. Overall, our findings as well as previous studies suggest that our discovered variants affect genes that have an important impact on neurogenesis.

Our study had a few limitations. First, it had a relatively small cohort size. This necessitates further study with a larger sample size for increased statistical power. We also only examined variants with a simple segregation pattern and limited our analysis to interactions among variants. With a large sample size, methods incorporating variants with incomplete penetrance (i.e., not following a strict segregation pattern) could further improve the power of the analysis. Expanding the region of analysis beyond miRNA seed regions paired with tools to predict gain or loss of binding sites such as SBSA [52] could also aid in identifying additional pathogenic variants in future works.

Psychiatric disorders in general have a large polygenic component [53], with variants distributed throughout the genome with varying effect sizes [54]. The genetic overlap between psychiatric disorders is considerable but not total. The importance of understanding the degree and specificity of overlap between disorders and closely related phenotypes is multifaceted, including implications of homogeneity in recruiting subjects for clinical research, understanding shared genetic risk, and for biological studies across disease contexts. Here, we focused on ASD, ADHD, LI, RI, and the SRS. Evaluating the relationship between ASD, LI, and RI was among the main goals of the NJLAGS study, with recruiting choices intended to identify families with high genetic burden for both ASD and language impairments (both spoken, LI, and written, RI) [11,55,56,57,58]. The SRS is a quantitative scale that captures a key component of the ASD phenotype, since social skills are a pillar of diagnosis [59]. To date, the relationship between language (LI/RI) and ASD has shown to be complex [11,58], with this study further showing indications of the polygenic nature of that relationship. ADHD is well known to be genetically related to RI, but again, when applied to families selected for ASD, the relationship is complex and requires further studies [60].

## 5. Conclusions

In conclusion, our study identified a number of high-confidence segregating variants that could affect the miRNA/mRNA regulation pathway. Many candidate genes and pathways play important roles in neuronal development and could contribute to the etiology of ASD and associated disorders. Previous studies have identified non-coding regulatory mutations associated with ASD. While our study did not replicate the findings from these studies [9,10], our results support the miRNA regulation pathway as an important contributor to the etiology of ASD and related NDDs. Future studies with experimental functional validation could further elucidate the roles of our identified genes in the etiology of ASD and related NDDs.

## Figures and Tables

**Table 1:** Summary of Samples and Families.

	Patients	Male	Female	Families	Dominant	Recessive/*de novo*
**ASD**	83	65	18	73	0	67
**LI**	117	86	31	73	9	58
**RI**	134	96	38	73	9	42
**SRS**	83	60	23	59	25	43
**ADHD**	63	43	20	47	10	24
**All Samples**	272	166	106	73	35	67

The first three columns are the total number of affected individuals, male, and female affected individuals, respectively. Families indicate the total number of families that contain at least one affected individual with the respective phenotype. Dominant and Recessive/*de novo* are the number of families that meet the criteria for the specific mode of inheritance.

**Table 2:** miRNA candidate variants.

miRNA	Region	Target Genes	chr	pos	rsID	Ref	Alt	AF_2_exome	AF_2_genome	AF_3	Family	Inheritance	Phenotype
hsa-miR-6780a-3p	seed	179	chr17	40860121	rs200279579	A	G	1.23 × 10^−4^	9.42 × 10^−5^	1.26 × 10^−4^	FAM5	dominant	RI
hsa-miR-1225-5p	mature	43	chr16	2140269	rs371749301	C	A	1.48 × 10^−5^	4.72 × 10^−5^	2.00 × 10^−4^	FAM58	dominant	RI
hsa-miR-2277-3p	mature	37	chr5	92956416	rs550720421	G	T	4.62 × 10^−4^	4.24 × 10^−4^	8.83 × 10^−4^	FAM58	dominant	RI
hsa-miR-548j-5p	mature	42	chr22	26951249	rs565141718	C	T	3.95 × 10^−4^	4.70 × 10^−5^	1.41 × 10^−4^	FAM13	dominant	LI
hsa-miR-100-5p	mature	26	chr11	122022992	rs761222509	G	A	1.93 × 10^−5^	N/A	7.42 × 10^−6^	FAM66	dominant	RI

miRNA: HGNC Symbol. Region: miRNA region. Target Genes: the number of miRNA target genes predicted by TargetScanHuman. chr: Chromosome. pos: Variant position. rsID: DBSNP150 reference SNV number. Ref: Reference allele. Alt: Alternate Allele. AF_2_exome: Population AF from gnomAD v2 non-neuro exome database. AF_2_genome: Population AF from gnomAD v2 non-neuro genome database. AF_3: Population AF from gnomAD v3_non_neuro database. Family: Affected Family ID. Inheritance: Inheritance mode for the affected family. Phenotype: Phenotype of the affected individual.

**Table 3:** Summary of 3′UTR Candidate Genes. Total number of genes found in each phenotype and inheritance pattern.

	ASD	ADHD	LI	RI	SRS	Total Unique
Dominant (AF < 1%)	0	26	30	79	33	138
Recessive (AF < 1%)	3	2	3	3	2	5
*de novo* (AF < 0.1%)	9	4	6	5	4	10
Total Unique	12	32	39	87	39	152

## Data Availability

The raw sequencing reads, variants, and genotypes for all samples are available in the National Institute of Mental Health Data Archive (NDA) under experiments C1932 and C2933.

## Supplementary Materials

The following are available online at https://www.mdpi.com/article/10.3390/genes13081329/s1, Table S1: Summary of Family Pedigrees; Table S2: Previously implicated NDD Genes; Table S3: Summary of miRNA variants; Table S4: Summary of 3_UTR variants; Table S5: Summary of Target Genes; Table S6: Candidate Gene GO Analysis; Table S7: Summary of 35 Genes in Figure 4; Table S8: GO Analysis of Genes in Figure 4; Table S9: Summary of Network Interactions in Figure 4; TableS10: Summary of Network Interactions in Figure 5.
